# The Steady-State Serum Concentration of Genistein Aglycone Is Affected by Formulation: A Bioequivalence Study of Bone Products

**DOI:** 10.1155/2013/273498

**Published:** 2012-12-31

**Authors:** Alessandra Bitto, Bruce P. Burnett, Francesca Polito, Silvia Russo, Rosario D'Anna, Lakshmi Pillai, Francesco Squadrito, Domenica Altavilla, Robert M. Levy

**Affiliations:** ^1^Department of Clinical and Experimental Medicine and Pharmacology, University of Messina, 98125 Messina, Italy; ^2^Department of Medical Education and Scientific Affairs, Primus Pharmaceuticals, Inc., Scottsdale, AZ, USA; ^3^Section of Physiology and Human Nutrition, Department of Biochemical, Physiological and Nutritional Sciences, University of Messina, Messina, Italy; ^4^Department of Obstetrical and Gynecological Sciences, University of Messina, Messina, Italy; ^5^Department of Clinical Affairs, Primus Pharmaceuticals, Inc., Scottsdale, AZ, USA

## Abstract

An FDA-regulated, prescription medical food (Fosteum; 27 mg natural genistein, 200 IU cholecalciferol, 20 mg citrated zinc bisglycinate (4 mg elemental zinc) per capsule) and an over-the-counter (OTC) supplement (Citracal Plus Bone Density Builder; 27 mg synthetic genistein, 600 mg elemental calcium (calcium citrate), 400 IU vitamin D_3_, 50 mg magnesium, 7.5 mg zinc, 1 mg copper, 75 **μ**g molybdenum, 250 **μ**g boron per two tablets) were compared to a clinically proven bone formulation (27 mg natural genistein, 400 IU cholecalciferol, 500 mg elemental calcium (calcium carbonate) per tablet; the Squadrito formulation) in an 8-day steady-state pharmacokinetic (PK) study of healthy postmenopausal women (*n* = 30) randomized to receive 54 mg of genistein per day. Trough serum samples were obtained before the final dose on the morning of the ninth day followed by sampling at 1, 2, 4, 6, 8, 10, 12, 24, 36, 48, 72, and 96 hrs. Total serum genistein, after **β**-glucuronidase/sulfatase digestion, was measured by time-resolved fluorometric assay. Maximal time (*T*_max_), concentration (*C*_max_), half-life (*T*_1/2_), and area under the curve (AUC) were determined for genistein in each formulation. Fosteum and the Squadrito study formulation were equivalent for genistein *T*_max_ (2 hrs), *C*_max_ (0.7 *μ*M), *T*_1/2_ (18 ± 6.9 versus 21 ± 4.9 hrs), and AUC (9221 ± 413 versus 9818 ± 1370 ng*·*hr/mL). The OTC supplement's synthetically derived genistein, however, showed altered *T*_max_ (6 hrs), *C*_max_ (0.57 **μ**M), *T*_1/2_ (8.3 ± 1.9 hrs), and AUC (6474 ± 287 ng*·*hr/mL). Differences in uptake may be due to multiple ingredients in the OTC supplement which interfere with genistein absorption.

## 1. Introduction

**Figure 1:**
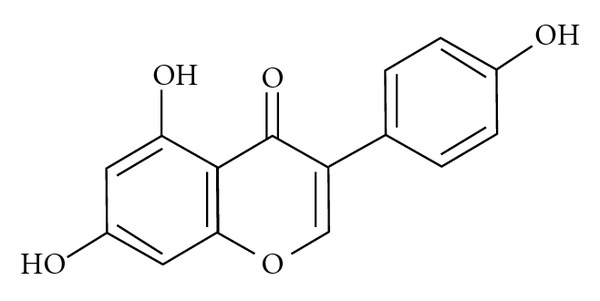
Genistein aglycone.

Asian populations consume ~25–50 mg of isoflavones daily with 10% consuming more than 100 mg per day [1]. Americans, on the contrary, consume ~0.15–3 mg per day [2, 3]. Much of the isoflavone consumed by Asian populations is in the form of aglycone from fermented soy product rather than glycoside forms consumed in mostly processed food in the USA. Many epidemiological studies of Asian women support an inverse relationship between isoflavone intake and bone loss as well as fracture rate. A large prospective study of 24,403 Chinese postmenopausal women, for example, demonstrated that ≥21 mg daily soy isoflavone consumption dramatically reduced subsequent fracture incidence over a 4.5-year period [4]. Most clinical trials, especially in the USA, are performed on extracted glucoside isoflavones from soy rather than aglycones forms which are found in fermented foods such as tofu, miso, and natto in the Asian diet. Recent clinical trials of 120 mg/day mixed glycoside isoflavones given to healthy postmenopausal women for 2 and 3 yrs, however, showed only modest effects on bone metabolism [5–7]. In a well-controlled dietary trial, natto, a fermented soy product containing 35 mg aglycone isoflavones enriched with 3.6 mg zinc given twice daily, showed statistically significant increases bone formation and decreases bone resorption markers over natto alone [8]. To date, only genistein (aglycone), as a single entity, has been tested in well-controlled clinical trials for its effectiveness on building bone (Figure 1) though studies have begun on S-equol, the intestinal bacterial conversion product of daidzein, for its effect on bone makers [9].

In a 12-month randomized, placebo-controlled clinical trial (*n* = 90), 54 mg of genistein administered daily showed equivalent increases in femoral neck and lumbar spine bone mineral density (BMD) (+3%) compared to the group given 1 mg of 17*β*-estradiol/0.5 mg noresthisterone acetate per day while the placebo group BMD declined [10]. All groups also received calcium carbonate (1000 mg) and cholecalciferol (800 IU) per day. This pilot study result was replicated in a larger (*n* = 389), long-term (24 months) study using the same amount of genistein compared to placebo [10]. A subcohort (*n* = 138) of this initial study extended to 36 months showed a similar rate of BMD increase (~3%/yr to ~9% over 3 yrs) at femoral neck and lumbar spine while the placebo BMD decreased by ~10-11% at the femoral neck and lumbar spine. [11]. Markers of bone formation increased substantially while markers of bone resorption decreased significantly for the genistein groups in these studies [10–13]. Bone quality assessed by quantitative ultrasound from the subcohort had statistically increased speed of sound, bone transmission time, and stiffness indices versus placebo [14]. In addition, a bone structural study in ovariectomized rats with established osteoporosis in which genistein was compared to alendronate, raloxifene, estradiol, and placebo showed superiority of genistein for all bone formation indices, fracture resistance, and histology (both trabecular and cortical bone) compared to all other therapies [15]. These results have spawned the development of products for bone loss containing pure genistein but no comparative studies have been performed between these new commercial products.

Bioavailability comparisons can predict whether certain active ingredients in product formulations will show the same effect in clinical trials. It has been established that glycoside isoflavones are poorly absorbed in the intestine and that hydrolysis of the glycosidic bond by *β*-glucosidases activates the aglycone for rapid absorption across the intestinal wall [16–18]. Most isoflavone bioavailability studies are performed in a food matrix using fermented or nonfermented products. Pure genistein and its glucoside, genistin, have been compared for uptake and the appearance in plasma as well as excretion of phase II metabolites in urine of healthy young women after multiple doses [19]. This study showed that there were differences in total genistein *C*_max⁡_ and AUC as well as several genistein metabolites. The addition of purified components in combination with genistein or genistin is not well studied. One recent study showed that the continuous administration of fructooligosaccharide, a prebiotic, dramatically changed genistein and daidzein *C*_max⁡_ and AUC obtained from a soy powder containing primarily genistin and daidzin [20]. With these data in mind, it is important to perform bioavailability comparisons for formulations containing purified active ingredients and excipients which surround isoflavones before testing them clinically.

The FDA-regulated, prescription medical food [21], Fosteum, for the clinical dietary management of osteopenia and osteoporosis under physician supervision was formulated in collaboration with Squadrito and coworkers as previously described [11, 12]. The OTC bone supplement, Citracal Plus Bone Density Builder, is based on a bone support formula already on the market (Citracal) and uses literature support to justify the addition of genistein [11, 12]. Since the Squadrito formulation is the only mixture which contains genistein that has been clinically proven to build bone, the first step in determining whether Fosteum and/or Citracal Plus Bone Density Builder are bioequivalent is to test the bioavailability of genistein. Therefore, the steady-state pharmacokinetics of 54 mg of genistein per day was compared for the Squadrito formulation to that of the Fosteum and Citracal Plus Bone Density Builder. 

## 2. Materials and Methods

### 2.1. Materials

The genistein in the prescription medical food (27 mg natural genistein, 200 IU cholecalciferol, 20 mg citrated zinc bisglycinate (4 mg elemental zinc) per capsule) (Fosteum, Primus Pharmaceuticals, Inc.) and the Squadrito study formulation (27 mg natural genistein, 400 IU cholecalciferol, 500 mg calcium (carbonate salt) per tablet) are obtained from natural sources, whereas in OTC supplement (13.5 mg synthetic genistein, 300 mg calcium (as citrate and carbonate salts), 200 IU vitamin D_3_, 25 mg magnesium (as stearate, oxide, and silicate salts) 3.75 mg zinc (oxide salt), 0.5 mg copper (gluconate salt), 1 mg manganese (gluconate salt), 37.5 *μ*g molybdenum (amino acid chelate), 125 *μ*g sodium borate per tablet) (Citracal Plus Bone Density Builder, Bayer HealthCare LLC) genistein is synthetically produced. All mineral levels are expressed in elemental mass units. All three products purport genistein purity of ~99%. The compositions and daily dosages of each formulation tested in the PK study are shown in Table 1.

### 2.2. Analysis of Genistein Content and Purity in Study Products

In order to compare the purity of genistein and minor isoflavones in each product, HPLC analysis was performed [22]. Briefly, samples were pulverized and then vortexed for 1 min in 2.5 mL of 1 : 1 : 1, hexane to methyl tert-butyl ether to methylene chloride extraction solvent. The samples were then vortexed gently for 15 min followed by a 10 min centrifugation at 3000 rpm to separate the aqueous and organic layers. The aqueous layer of each sample was then frozen at −80°C and the organic layer poured into a 10 mL glass conical screw cap tube where the sample was dried with nitrogen gas at 40°C.

The dried extracts, as well as separate controls (genistein, daidzein, glycitein, and their glycosides), were reconstituted with 0.2 mL of 1 : 1, mobile phase buffer A (0.05% formic acid and 5 mM ammonium formate in distilled water) to mobile phase buffer B (0.05% formic acid and 5 mM ammonium formate in an 80 : 10 : 10 ratio, acetonitrile to methanol to distilled water). Samples were vigorously vortexed for 5 min and then centrifuged for 2 min at 1500 rpm to remove any insoluble material. The supernatants were removed and transferred to 0.25 mL polypropylene injection vials with caps for each chromatography run. Areas under curves were compared to standards to obtain purities.

### 2.3. Subjects

After the Ethical Committee approved the study, a total of 30 participants were recruited among those reporting to the Center for Menopause in the Department of Obstetrical and Gynaecological Sciences at the University of Messina (Messina, Italy). All participants gave informed consent. All women were 50–65 yrs old, had been postmenopausal for at least 12 months at baseline and were in good general health. At the start of the study, a complete medical and family history was obtained. Exclusion criteria were the same of our previously published reports [12].

### 2.4. Diet

The intake of soy products, legumes, or other nutrient supplements which could contain isoflavones was prohibited for the 2 weeks before and during the study. The isoflavone intake before randomization as assessed by a food-frequency questionnaire was 1 to 2 mg/day. This intake has been shown to be typical of Western populations.

### 2.5. Treatment Protocol

The PK study was carried out at the laboratory of the Section of Pharmacology, Department of Clinical and Experimental Medicine and Pharmacology, University of Messina. Participants were randomly assigned to receive one of the following products for orally 8 days: 1 capsule twice daily (BID) of the medical food (*n* = 10); 1 tablet BID of the Squadrito study formulation (*n* = 10); or 2 tablets BID of the OTC supplement (*n* = 10). On the morning of the ninth day, trough serum samples (basal, 0 hr) were obtained following which subjects were given their final dose of study product. Blood samples were then collected using an intravenous cannula at 1, 2, 4, 6, 8, 10, 12, 24, 36, 48, 72, and 96 hrs after final dosing. All other forms of calcium or vitamin D_3_ were proscribed before and during the study.

The maximal plasma concentration (*C*_max⁡_, nmol/L) and time to maximal plasma concentration (*T*_max⁡_, hr) were obtained directly by the visual inspection of each subject's plasma concentration-time profile. The areas under the plasma concentration-time curve (AUC, ng·hr/mL) as well as half-life (*T*_1/2_, hrs) were determined for each formulation by using the PK Solutions 2.0 software.

### 2.6. Plasma Genistein Levels

Total genistein levels were measured in plasma samples by a time-resolved fluorometric assay following the manufacturer's instructions (TR-FIA test; Labmaster, Turku, Finland). Briefly, 200 *μ*L of 100 mM acetate buffer (pH 5.0) containing 0.2 U/mL *β*-glucuronidase and 2 U/mL sulfatase was added to 200 *μ*L serum. Samples were then incubated overnight at 37°C. After incubation, free genistein was extracted twice with 1.5 mL diethyl ether by mixing for 3 min. The water phase is frozen in dry ice-ethanol mixture, and the ether phase was transferred into a disposable glass tube. After thawing, the water phase was reextracted with ether, and the ether phases are combined and evaporated to dryness at 45°C water bath. Then, 200 *μ*L assay buffer was added to each sample. A 20 *μ*L aliquot of this solution was used for time-resolved fluoroimmunoassay. The fluorescent signal was read using a Perkin-Elmer (Norwalk, CT) Victor 1420 multilabel counter.

### 2.7. Statistical Analysis

Total plasma genistein concentrations were obtained at each time point in duplicate for each subject and PK analyses were performed. The primary variables of interest were *C*_max⁡_ (the maximum observed concentration of total genistein), *T*_max⁡_ (the elapsed time at which *C*_max⁡_ was observed), *T*_1/2_ (the elapsed time at which genistein concentration was half of *C*_max⁡_), and the imputed area under the curve (AUC) estimating the total body exposure to genistein over time. Area under the curve was computed by interpolating the concentrations of total genistein in the intervals between recordings using trapezoid calculations. Imputation was performed by using cubic spline estimation. Each of these variables was computed for each participant, and mean values and standard deviations were computed for the sample. Any value exhibiting *a* > 3 standard deviations (*n* = 3) from the mean were removed from each analysis. A student's *t*-test was conducted for each measure to see if the observed difference in means was significant. Descriptive statistics were presented for each of the primary outcome variables.

## 3. Results

### 3.1. Genistein Content and Purity in Study Products

**Figure 2:**
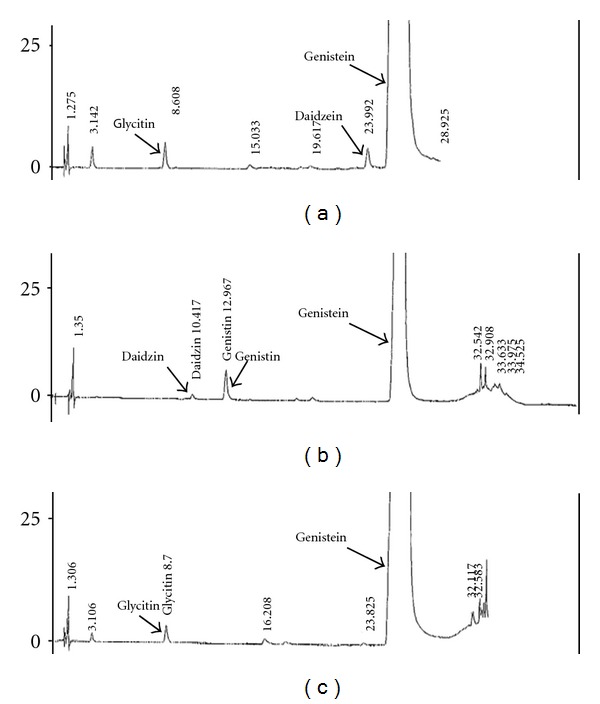
High performance liquid chromatography (HPLC) comparison of genistein purity extracted from the medical food product indicated for osteopenia/osteoporosis (a), the Squadrito study formulation (b), and the OTC bone supplement (c).

The genistein in both the prescription medical food and the Squadrito study formulation are from natural sources, whereas the genistein in the OTC supplement is produced synthetically. The mineral content for all products was confirmed by nutritional analysis (data not shown). HPLC analysis shows that the genistein molecules extracted from each formulation have equivalent purity with relative minor impurities of other isoflavone(s) amounting to <1% (Figure 2). When the chromatograms are aligned and enlarged to compare the very small differences in genistein content between the two natural sources in the medical food (Figure 2(a)) and the Squadrito study formulation (Figure 2(b)) to that of the synthetic source in the supplement (Figure 2(c)), there are only small differences in the contaminating isoflavones of all products. Fosteum is contaminated by a small amount of glycitin, and daidzein, the Squadrito study formulation contains daidzin and genistin and the Citracal Plus Bone Builder supplement has a small amount of glycitin. No appreciable difference is seen in genistein purity in any of the three products with other aglycone impurities being less than 1%. Since the genistein levels are equivalent in all three products, a PK study of genistein should reveal any differences in uptake or excretion based on the surrounding vitamin, mineral, and excipient content in each formulation. Thus a bioavailability analysis can determine if genistein is bioequivalent in Fosteum and/or Citracal Plus Bone Density Builder compared to the Squadrito formulation which has been tested in clinical trials on bone.

### 3.2. Pharmacokinetic Comparison of Plasma Genistein in Each Treatment Group

**Figure 3:**
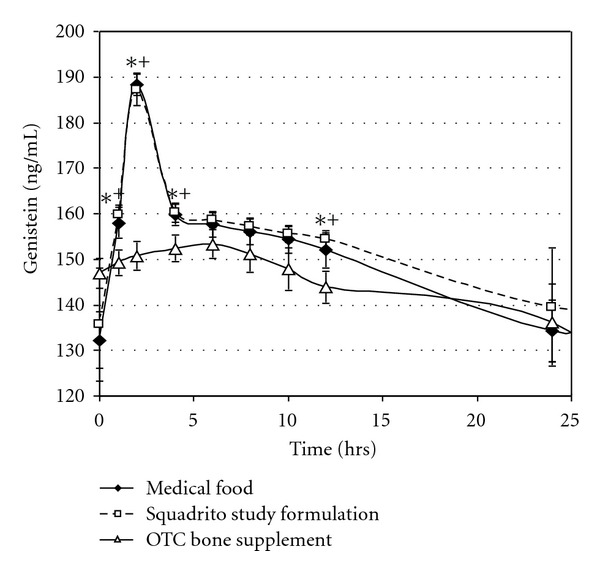
The pharmacokinetic profile for the first 24 hours after the last dose of each study product obtained after 8 days 54 mg per day intake of the medical food indicated for osteopenia/osteoporosis (*♦*), the Squadrito study formulation (□) and the OTC bone supplement (Δ). *P < 0.05 for the medical food versus the OTC bone supplement, ^+^ *P* < 0.05 for the Squadrito study formulation versus the OTC bone supplement.

The PK profile for genistein obtained during the first 24 hours after the last dose of each study product standardized to 54 mg per day after 8 days intake is shown in Figure 3. Genistein from the medical food and the Squadrito study formulation were absorbed and excreted at approximately equal rates with statistically significant higher concentrations at 1, 2, 5, and 12 hrs. The genistein contained in the supplement showed a much lower overall uptake by comparison. The PK analysis reinforced in this plot showed that the *T*_max⁡_ of genistein for both the medical food and the Squadrito study formulation occurred 4 hrs earlier than that found in Citracal Plus Bone Density Builder supplement and the genistein *C*_max⁡_ was also ~23% higher at this point (Table 2). 

The medical food and the Squadrito study formulation genistein peak serum concentrations are very similar with only nonstatistical differences in concentration at each time point over the course of the terminal half-lives for the products. This would represent the normal PK profile before a subsequent dose was consumed. The absorption and depletion profiles of genistein from the medical food and the Squadrito study formulation exactly overlapped during the initial phase lasting approximately 5 hrs. When compared to OTC supplement, the medical food had a 42% greater AUC while the Squadrito study formulation had a 52% greater AUC for genistein over the entire 96 hr time course suggesting dramatic differences in steady-state genistein absorption. Even after the 1 and 2 hr time points, the steady state amount of genistein found in the serum was significantly lower from the supplement compared to the medical food and the Squadrito study formulation suggesting interfering ingredients within the supplement.

## 4. Discussion

Health benefits of isoflavones are directly related to their bioavailability. Bioavailability is dependent upon an individual's state of health, intestinal bacterial flora, sex, age, food matrix in which isoflavones are consumed, the mix of isoflavones in products as well as host genetics [23]. The results of this PK analysis of three different bone formulations show genistein absorption is affected by specific ingredients formulated with the isoflavone which could have clinical implications on efficacy (Table 2; Figure 3). There are a multitude of factors which could account for this difference. 

Normally, genistein is freely absorbed from the intestine and a large fraction is converted to 7*β*-O-glucuronide as it crosses the brush border and ultimately enters the portal vein [24]. Intestinal bacteria are known to influence glucuronidation and may also drive sulfonation [25, 26]. Only a small percentage of the parent molecule remains as free genistein once it reaches the liver. Once in the liver, genistein undergoes additional biotransformation via CYP450-mediated hydroxylation [27] followed by glucuronidation and sulfation by UDP-glucuronosyl transferase and sulphotransferases, respectively [24]. A large majority of glucuronidated genistein undergoes efficient enterohepatic recirculation following biliary excretion. The preponderance of circulating genistein in serum has been found to be in the form of glucuronidate and sulfate conjugates [28].

Food, isolated nutrient molecules and binders, when coadministered with drugs, are known to affect their absorption, distribution in the body, metabolism in the lumen, liver and cells, and elimination [29]. This issue is so important that the FDA has issued guidance on oral administration of drugs with food, their bioavailability and need for bioequivalence studies to assure proper guidance for administration of therapeutic compounds [30]. Genistein is considered a class 2 compound with low solubility and high permeability by the FDA's Biopharmaceutics Classification System. Though there are no formal requirements for this type of analysis of medical foods or supplements, it is important that fasting and fed PK and bioequivalence studies be performed, especially since medical foods have a statutory requirement to be indicated for a specific disease and must be administered under the direction of a physician [21]. Indeed, a fasting and fed PK study of genistein has been performed on the medical food Fosteum indicated for osteopenia/osteoporosis suggesting a minor, nonstatistical effect of food on absorption [31]. There is no published data on genistein bioavailability from the OTC Citracal Plus Bone Density Builder supplement. This steady-state PK study demonstrates that the medical food product for osteopenia/osteoporosis is equivalent to the clinically proven Squadrito study formulation for absorption and bioavailability of genistein, whereas the OTC supplement formulation dramatically and statistically affects the isoflavone absorption (Table 2; Figure 3).

Absorption of bioactive substances is influenced by several different factors such as the intestinal solubility and permeability [32]. Another factor that can affect absorption of bioactive molecules is viscosity induced by food additives, such as guar gum [33]. Citrate, an approved food additive, is also known to increase viscosity in the presence of collagen and fibrous material [34] as well as change the water absorption profile in different parts of the small intestine [35]. It has been added to different oral rehydration formulations to modulate acidosis and glycemic index as a viscosity-promoting agent [36–38]. Based on the above data, it is possible that normal dietary fiber in those randomized to the bone supplement group had increased gastrointestinal viscosity during the time of dosing which affected genistein uptake due to the dissociation of the citrate and calcium ions in the stomach. Though Fosteum contains citrated zinc bisglycinate, citrate along with glycine tightly coordinate zinc and is not ionized in the stomach. Preclinical studies have shown that the zinc from chelates is dissociated from the coordinating molecules on the lumen of the intestine [39]. Hence, the chance of the citrate portion of the chelate interacting with dietary fiber is minimal. Other mechanisms may also account of the lower level of genistein absorption from the supplement.

ATP-binding cassette (ABC) transport proteins are responsible, in part, for the transport of flavonoids, including isoflavones, into luminal intestinal cells for absorption [40]. Genistein specifically interacts with the ABCG2 receptor in a variety of cells including those in the intestinal lumen [41]. Calcium and magnesium ions are typically actively absorbed via transient receptor potential channel proteins (TRP) in the duodenum [42]. Vitamin D_3_ is needed for calcium uptake through these channels while magnesium serves as a cofactor for ABC transport proteins in the uptake of flavonoid molecules. There is no reported evidence that calcium, magnesium or other ions inhibit ABC receptors. Isoflavones, such as genistein, are also transferred from the intestine into the epithelial lumen by organic anion transport proteins (OATs) [43]. The OAT receptor family also serves to maintain anion balance throughout the body, including in the intestinal lumen [44] and is a subclass of a superfamily of proteins termed major facilitator superfamily (MFS) transporters [45, 46]. Citrate is known to interact with both OAT receptors [44] as well as with members of the MFS called citrate-H+ symporter (CitA) [47] and Na+/citrate transporters [48]. Another OAT receptor, Mrp2, also known as canalicular multispecific organic anion transporter (cMOAT) and ABCC2 binds organic anions like citrate and gluconate [49]. Both ABCC2 and ABCG2 have extensive homology and exist together in the intestinal lumen having a broad range of nutrient transport capabilities. These include the transport of organic anions, glucuronidated and sulfonated molecules, and a number of drugs [50]. These receptors have been shown to have specific functional overlap in absorption of various molecules [51]. Therefore, organic anions such as citrate, silicate, gluconate, and stearate present as counterions in the OTC Citracal Plus Bone Density Builder supplement formulation may directly compete with genistein for absorption on these receptors and transporters. This and the possibility that citrate increases viscosity, and hence slows gastric emptying, might explain the difference in uptake resulting in a lower *C*_max⁡_, lengthened *T*_max⁡_ and decreased AUC compared to the medical food product and the Squadrito study formulation. The carbonate anion in the Squadrito study formulation is known to interact with the solute carrier family (SLC) of receptors [52], rather than ABC or OAT receptors. This may explain why calcium carbonate does not affect genistein absorption while the calcium citrate supplement formulation appears to do so.

## 5. Conclusion

The medical food for osteopenia/osteoporosis, Fosteum, and Squadrito study formulation tested for bone building in clinical trials are bioequivalent for absorption of genistein compared to that from bone supplement Citracal Plus Bone Density Builder which inhibits genistein uptake. Even with the 10% difference in AUC between the medical food and the Squadrito study formulation over the 96 hr period, one could expect similar genistein pharmacokinetic behavior from both products under usual conditions of use. The steady-state genistein concentration attained by dosing with the OTC Citracal Plus Bone Density Builder supplement, however, would presumably be significantly lower compared to Fosteum and the Squadrito study formulation even over long periods of time. This difference could adversely affect overall efficacy on bone metabolism. Based on this evidence, care must be taken when combining bioactive substances like genistein with specific salts to prevent changes in viscosity or competition for receptors or transport proteins during intestinal absorption.

## Figures and Tables

**Table 1:** Composition of three formulations for bone, a medical food indicated for osteopenia/osteoporosis, the Squadrito study formulation, and the OTC bone supplement. All minerals are given as elemental mass.

Constituent	Medical food	Squadrito study formulation	OTC bone supplement
Dosage form	Capsule	Tablet	Tablet
Daily dose	2	2	4
Genistein aglycone	54 mg	54 mg	54 mg
Vitamin D_3_	400 IU	800 IU	800 IU
Calcium (elemental)	120 mg	1000 mg	1200 mg
Magnesium (elemental)			100 mg
Zinc (elemental)	8 mg		15 mg
Copper (elemental)			2 mg
Manganese (elemental)			4 mg
Molybdenum (elemental)			150 *μ*g
Boron (elemental)			500 *μ*g

IU: international units.

OTC: over-the-counter.

**Table 2:** The maximal plasma concentration (*C*_max⁡_), time to maximal plasma concentration (*T*_max⁡_), areas under curve (AUC) and half-life (*T*_1/2_) after steady administration of the medical food indicated for osteopenia/osteoporosis, the Squadrito study formulation, and the OTC bone supplement.

Study parameters	Medical food	Squadrito study formulation	OTC bone supplement
*T*_max⁡_ (hrs)	2	2	6
*C*_max⁡_ (ng/mL ± StDev)	188.4 ± 2.5	187.1 ± 3.5	153.3 ± 3.5
AUC (ng·hr/mL)	9221 ± 413	9818 ± 1370	6474 ± 287
*T*_1/2_ (hrs ± StDev)	18.0 ± 6.9	20.9 ± 4.9	8.3 ± 1.9
